# Artificial Intelligence in Obstetric Ultrasound: An Update and Future Applications

**DOI:** 10.3389/fmed.2021.733468

**Published:** 2021-08-27

**Authors:** Zhiyi Chen, Zhenyu Liu, Meng Du, Ziyao Wang

**Affiliations:** ^1^The First Affiliated Hospital, Medical Imaging Centre, Hengyang Medical School, University of South China, Hengyang, China; ^2^Institute of Medical Imaging, University of South China, Hengyang, China

**Keywords:** artificial intelligence, obstetric ultrasound, automatic measurement, segmentation, classification, ultrasound telemedicine

## Abstract

Artificial intelligence (AI) can support clinical decisions and provide quality assurance for images. Although ultrasonography is commonly used in the field of obstetrics and gynecology, the use of AI is still in a stage of infancy. Nevertheless, in repetitive ultrasound examinations, such as those involving automatic positioning and identification of fetal structures, prediction of gestational age (GA), and real-time image quality assurance, AI has great potential. To realize its application, it is necessary to promote interdisciplinary communication between AI developers and sonographers. In this review, we outlined the benefits of AI technology in obstetric ultrasound diagnosis by optimizing image acquisition, quantification, segmentation, and location identification, which can be helpful for obstetric ultrasound diagnosis in different periods of pregnancy.

## Introduction

Ultrasound is utilized throughout the entire process of pregnancy. It is critical in the observation of fetal growth and development as well as the diagnosis and treatment of disease. It can provide detailed information on fetal anatomy with high-quality images and improved diagnostic accuracy (1). At present, two-dimensional (2D) imaging and three-dimensional (3D) ultrasound are widely used to measure fetal structures, assess organ functions, and diagnose diseases (2, 3). Access to quality obstetric ultrasound imaging is important of accurate diagnosis and treatment. However, it is easily subjected to involuntary fetal movements at the early stage of pregnancy, and the structures of interest are almost always occluded at the second- and last trimester, which may cause difficulties for examination and mis-diagnosis. The subjectivity of measurements and observation were also influencing factors for the acquirement of high-quality ultrasound images, standard measurements, and precise diagnosis.

In the last decade, to reduce intra- and inter-observer measurement variations and to improve diagnostic accuracy, automatic measurements, and assessments based on artificial intelligence (AI) have been introduced (4, 5). The application of AI in accuracy improvement of obstetric ultrasound includes three aspects: structure identification, automatic and standardize measurements, and classification diagnosis (Table 1). Since obstetric ultrasound is time-consuming, the use of AI could also reduce examination time and improve workflow (6, 7). Although lots of AI-aided techniques and commercial software have been launched to provide high resolution imaging and precise measurement for obstetric ultrasound, majority of the related researches are still in early stages. Promotion of interdisciplinary communication is one of the important steps toward establishing validity and clinical applicability of AI algorithms (8). Here, we systematically review and discuss the application as well as the advantages and disadvantages of AI in obstetrics ultrasound. Since the concern of ultrasound examination varies in different phases of pregnancy, AI-aided obstetric ultrasound can be divided into first trimester and second- and last trimester. The future outlook of obstetric ultrasound like telemedicine or telediagnosis service and virtual reality (VR) learning are also be concerned. We believe that the cooperative effort of researches from various disciplines will facilitate the translation of algorithms to the clinical application for AI-aided obstetric ultrasound.

**Table 1:** The application of AI in accuracy improvement of obstetric ultrasound.

	**First trimester**	**Second- and last-trimester**
Structure identification	Detection of fetal limbs	Recognition of facial structure Recognition of abdominal organs Calculation of ventricular volume and the thickness of ventricular wall
Automatic measurement	Measurement of NT and crown-rump length automatically	Estimate the AC/HC automatically Estimate the volume of fetal head and its internal structures Estimate the volume of fetal stomach and bladder
Classification and diagnosis	Assessment of fetal development	Neurodevelopmental maturity prediction Diagnosis of growth restriction
		Diagnosis of Craniocerebral malformation
		Assessment of fetal lung maturity
		Diagnosis of congenital heart disease
		Evaluation and prediction of premature birth by cervical ultrasound
		Evaluation of fetal weight and gestational age

## Application of AI in First Trimester

### Automatic Assessment of Fetal Growth and Development

Assessment of fetal growth and development in first trimester is critical for the diagnosis and intervention of pregnancy complications such as premature delivery and low birth weight. The routine method of fetal growth assessment was to measure crown-rump length by two-dimensional ultrasonography (2D-US). However, there was subjective dependence on single-section measurements by 2D-US, which may show no significant difference in the crown-rump length between normal and abnormal fetuses in first trimester. Three-dimensional-image volume measurements may provide more information on fetal development compared with 2D-image measurements, except that 3D imaging was time-consuming and the volume measurements could be underestimated. For this reason, a semi-automatic 3D-image volume calculation method based on pixel extraction and point-of-interest detection has been introduced. Using a 12-week-old fetus, this method recognized the fetal contours and calculated the fetal volume, thereby verifying the effectiveness of 3D imaging for accuracy of identification (9). Even so, the semi-automatic algorithm was unable to identify irregular items, and some faults of segmentation required manual amendment. In a recent study, the volume measurements of 104 fetuses and fetal appendages in first trimester (10–14 weeks) were determined using a 3D convolutional neural network (CNN) algorithm to realize the synchronous fully automatic segmentation of multiple anatomical structures, including the fetus, gestational sac, and placenta (10). Another study proposed an image processing solution based on 3D ultrasound, which contained segmentation of the fetus, estimation of standard biometry views, automatic measurements of biometry, and detection of fetal limbs (11). Since the fetus and fetal appurtenances were closely related, these algorithms can lead to a more systematic and comprehensive assessment.

### Assessment of Nuchal Translucency Thickness

**Figure 1:**
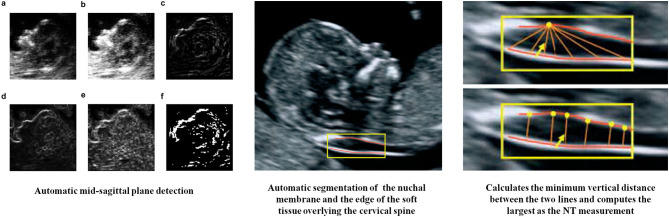
AI in measurement of NT thickness (12, 13). **(a)** Original image, **(b)** after histogram equalization, **(c–e)** detection results of different methods (directional edge image, SRAD edge image, and KAD edge image), **(f)** binary the direction edge image.

The measurement of NT thickness by 2D ultrasonography is critical for the detection of chromosomal malformations in the first trimester. The NT, which represents the maximal thickness between the fetal skin and the subcutaneous soft tissue at the level of the cervical spine, should be measured using a standard median sagittal image. However, this requires a high degree of accuracy and expertise. Due to small fetal structures, frequent fetal movements, and poor image quality, the measurement of the NT thickness usually requires multiple attempts. To decrease measurement errors and to reduce measurement difficulties, the fetal NT thickness was measured using semi-automated tools and the results were compared with manual measurements (12). In another study, the fetal NT thickness was measured using a standard median sagittal image combined with Deep Belief Networks that provided a prior knowledge for NT structure determination (13). Therefore, an automatic recognition model was constructed by combining sagittal plane information and complete 3D-image data, which achieved a detection accuracy of 88.6% (Figure 1).

## Application of AI-Aided Ultrasound in Second- and Last Trimester

### Fetal Head

Prenatal ultrasonography is critical for the assessment of fetal growth and development, detection of fetal abnormalities, and treatment of prenatal conditions to reduce the mortality rate. However, the fetal brain is one of the most difficult organs to evaluate by prenatal ultrasonography. The application of AI-aided ultrasound in the fetal brain consists of various aspects, such as fetal head biometry, measurement of cranial capacity, fully automatic segmentation of fetal head circumference (HC) and internal structure, and classification of normal and abnormal ultrasound features.

#### Automatic Segmentation of Fetal Head and Its Internal Structure

The HC is a major indicator of fetal growth, where abnormal values could indicate growth restriction. The measurement of this and other related indicators, such as the biparietal diameter, can also predict the gestational age (GA). In addition, prenatal ultrasonography can accurately assess craniocerebral development and detect intracranial malformations using multiple measurements and sections. However, the number of trained sonographers are inadequate in most developing countries, and inexperienced sonographers may find it difficult to obtain high-quality standard plane images, which can affect the determination of the GA and the assessment of fetal growth and development. Currently, the majority of ultrasonic machines have configured semi-automatic measurement software for the measurement of HC, which requires localization of two points (generally for the short diameter locating point on the section of biparietal diameter), and this may lead to measuring error. Previous studies have utilized various traditional methods to measure HC, such as randomized Hough transform, active contouring, etc. (6). In recent years, new methods were investigated based on deep learning methods (14). One of the studies have used the obstetric sweep protocol to achieve fully automatic analysis without the acquisition of a standard plane. The method was based on two full CNN s, where HC could be estimated from prior information, and GA could be determined by the curve of Hadlock. It was a great advancement that the errors made by standard plane scanning could be partly avoided (15). Another study had presented a CAD system based on random forest algorithm to automatically extract HC. The research included data from all trimesters, established the growth curve, and confirmed that evaluating results for each trimester separately was necessary, which was cost-effective and suitable for clinical settings which lacked experienced sonographers (16). On the other hand, to provide a basis of extraction of representative biometrics in the fetal head, Yang et al. proposed a fully automated solution to segment the whole fetal head based on 3D ultrasound, which achieved a Dice Similarity Coefficient of 96% (17). The research team further investigated a general framework for automatically segmenting fetal anatomical structures in 2D ultrasound images and thus made objective biometric measurements available. It showed a great ability on both the segmentation of fetal HC and abdominal circumference (AC) (18). Apart from HC, research was also concentrated on the accurate segmentation of internal structures of the fetal head. Common measurement items included fetal lateral ventricles, transcerebellar, cisterna magna, and posterior horn of the lateral ventricle, etc. (19, 20). The methods were not very different from those of neonates, except for the image quality (the fetal head was further from the ultrasound probe, and sheltered by adjacent tissue or anatomical structures) and the difficulties of location caused by the fetal activity. Most of these research was based on deep learning algorithms or other commercial software.

#### Automatic Recognition and Auxiliary Assessment of Fetal Facial Structures

Ultrasound imaging of fetal facial structures is routinely performed during prenatal ultrasonography to detect malformations. However, the usefulness of this method was limited by the expertise of sonographers. Imaging was also time consuming due to the length of time required for correct positioning. Furthermore, the limbs or umbilical cord could easily obstruct the facial structures. Although most facial deformities could be detected by 2D imaging with supplementation by 3D imaging if needed, it was still difficult to detect slight facial deformities. Artificial intelligence has an auxiliary role in fetal facial recognition, craniofacial development, and the detection of congenital malformations. In a previous study, the deep CNN method was applied to realize the automatic recognition of axial, coronal, and sagittal planes of the face, which effectively shortened the time of section recognition, and the recognition efficiency was 96.99% (21, 22). In another study, image registration technology was used to eliminate variations in fetal position, orientation, and size by taking the central areas of the head and eyes as feature points. Subsequently, craniofacial structures were automatically delineated using the segmentation method, and five craniofacial diameter lines (biparietal diameter, occipito-frontal diameter, interorbital diameter, bilateral orbital diameter, and orbital-calvaria diameter) were accurately measured to realize the intelligent diagnosis and evaluation of the fetal face (23).

**Figure 2:**
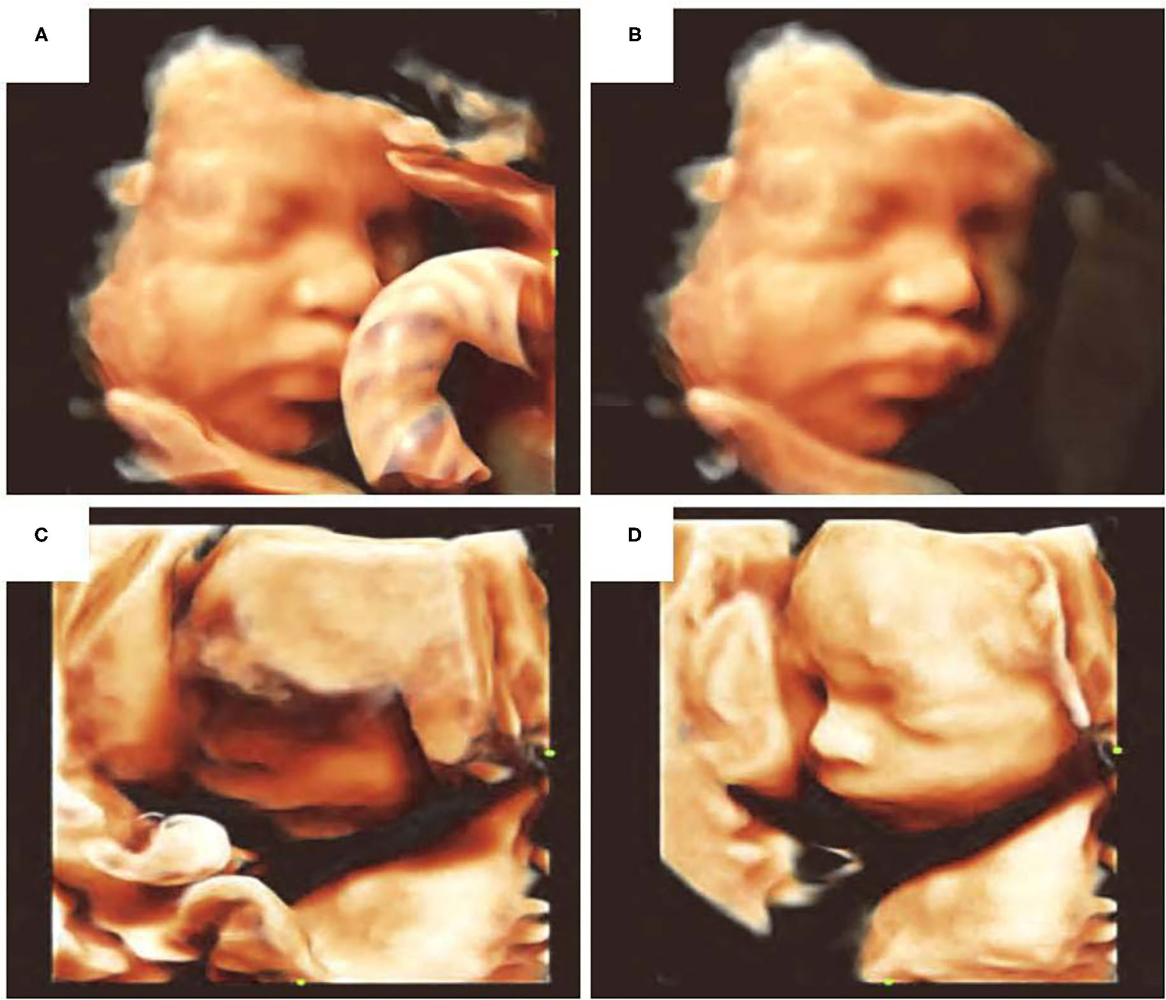
Optimization of fetal facial 3D ultrasound imaging. **(A)** Facial occlusion with umbilical cord, **(B)** elimination of facial occlusion, **(C)** facial edge incomplete reconstruction, **(D)** facial imaging optimization.

In addition to assessing facial development and malformations, AI can also improve diagnostic efficiency through pre-processing of the ultrasound images. For example, the automatic fetal face navigation system Smart Face (Resona 7, Mindary, Shenzhen, China) could automatically recognize the main facial features in 3D-image volume measurement data, as well as remove facial occlusions and optimize visual angles with one click, thereby rendering 3D imaging to be fast, convenient, and effective. Optimization involves acquisition of multi-frame sections of the fetal head image, facial edge detection through AI, recognition of facial contours, and elimination of facial occlusion to obtain a 3D image of facial contours. The fetal face model could also be used to calculate the direction of the face. It could also be rotated to a desired angle for further analysis (Figure 2).

#### Automatic Recognition of the Fetal Brain for the Diagnosis and Prognosis of Related Diseases

The final goal of AI in the medical field is to aid diagnosis and treatment. Image segmentation and recognition is an effective way to improve the reliability and accuracy of ultrasonography. In the diagnosis of growth restriction, 3D image of the fetal brain was produced using artificial boundaries and the fetal craniocerebral volume (including the cerebellum, brain, and frontal region) was measured. It was reported that the craniocerebral volume of abnormally small fetuses was smaller than that of normal fetuses. However, this study cannot reach fully automatic cerebral segmentation and the recognition of the shape (24). Due to the inaccurate prediction of the GA, which depended on the expertise of sonographers, differences in individual skull size due to ethnicity and the subjective acquisition of 2D diagnostic planes, another study constructed a semi-automated learning-based framework to identify age-related areas and signs from fetal craniocerebral ultrasound sections and associated them with neurodevelopmental maturity to provide new indicators of fetal development by simply observing craniocerebral morphological changes by ultrasonography (25). To make the evaluation more systematic, a study has created a classification tool for the diagnosis of intrauterine growth restriction. Besides the features of HC and GA, AC, BPD, and femur length (FL) were also used for ANN classification. This approach could help solve the problems of inter-and intra-observer variability (26). Recently, the application of AI in the diagnosis of fetal craniocerebral malformations is gradually increasing. Such advanced techniques have been loaded onto commercial ultrasound equipment, where “Smart plan” (Resona 7, Mindary, Shenzhen, China) is one of the representations. It could not only achieve automatic recognition of craniocerebral internal structures, but also helped with the diagnosis of diseases (27). It is possible for the system to use these measurements and recognition information to diagnose partial agenesis of the corpus callosum (identifying its structure), Dandy-Walker malformation (DWM), and other cerebellum-related diseases. Recent research has reported combinational algorithms (algorithm based on U-net for segmentation, and algorithm based on VGG-net for classification) for the diagnosis of fetal brain diseases, including ventriculomegaly, hydrocephalus, Blake pouch cyst (BPC), DWM, and cerebellar vermis hypoplasia (CVH). It showed that these algorithms were helpful for craniocerebral region segmentation, classification, and lesion localization in ultrasound diagnosis of fetal brain abnormalities, which might improve the diagnostic efficiency of for junior doctors (28).

### Accurate Recognition and Measurement of Fetal Abdominal Structures

The AC is the most valuable predictor of fetal weight. Standard abdominal sections can be used to determine the AC and evaluate fetal growth and development. Due to the low contrast of the fetal abdomen in acoustic images and the presence of irregular abdominal echoes, the measurement of the AC is more challenging compared to that of the HC. However, in applications involving AI, the identification of the abdomen and the measurement of the AC were similar to those of the head, and the main differences were in the anatomical structures to be identified (i.e., the presence, position, direction, and distance of nearby organs such as the spinal cord, gallbladder, and stomach) and the effect of the amniotic fluid sac on boundary recognition (29). Chen et al. reduced model overfitting through transfer learning and established a recognition system based on deep learning to standardize measurements of the AC. The model could also realize the automatic positioning of standard abdominal sections in the ultrasound dynamic image and the measurement of the fetal AC. Based on this, another study has divided the whole process into initial AC estimation, measurement, and plane acceptance checking (30). Every process consisted of a CNN and U-Net, which improved the accuracy of measurements and location. Shortcomings in image recognition and automatic measurement have been overcome by repeated updates of the algorithm and data volume amplifications. On the other hand, based on the limitation that 2D-image measurements of the long diameter and area could not recognize the viscera, 3D reconstruction can help to measure the volume of the hollow viscera. Therefore, presently, this method is used to measure the fetal stomach and bladder.

### Quantitative Texture Analysis of Fetal Lung Ultrasound Images

Poor lung development is the most common cause of prematurity and neonatal death. Although GA is one index of fetal lung maturity, assessment using GA alone has obvious individual variance. Amniotic fluid can provide information on the ratio of lecithin to sphingomyelin with high accuracy; however, this method involves puncture and is invasive. Therefore, it is critical to identify non-invasive methods that can accurately quantify fetal lung maturity. Recently, the application of ultrasonography in the assessment of fetal lung maturity has attracted attention. Generally, the evaluation of fetal pulmonary maturity using ultrasonography was obtained by comparison with the echogenicity of the liver, intestine, and placenta. However, it has a low inter-observer agreement and the diagnostic accuracy of this approach was not very acceptable. Based on this, texture analysis has been useful in the evaluation of fetal lung maturity by ultrasound. For example, Automatic Quantitative Ultrasonic Analysis (AQUA) software was reported to obtain texture images of the fetal lung in DICOM format, analyze the features, extract 30 features with the best correlations, and verify the correlations between features and GA (31). Lung texture was not affected by region-of-interest positioning, lung size, lung orientation, and the specific ultrasonic instrument or its frequency, and an image of another area was not required as a reference. Therefore, the feasibility and repeatability of the method was acceptable. In another study using the AQUA software, the sensitivity, specificity, and accuracy of software analysis in assessment of fetal pulmonary maturity were 95.1, 85.7, and 90.3%, respectively, compared with the lecithin-sphingomyelin ratio (32). Other researchers also established a similar system of texture analysis (e.g., Quantus^FLM^ for quantitative ultrasound analysis of fetal lung maturity) to identify preterm fetal lungs or predict the occurrence of neonatal respiratory diseases (33, 34). By texture heterogeneity analysis, it was reported that the heterogeneity of fetal lungs on ultrasound images in premature infants was decreased, whereas the heterogeneity of fetal lungs in full-term infants was increased, which was conducive to the early diagnosis of fetal lung dysplasia. Also, the feasibility of these software was validated in different clinical situation including proximal/distal lungs and US machines of different brands, etc. (35). The common thread was that the analysis was not affected by the adjustment of the gray value of the instrument. The system also had good applicability. However, limitations such as the limited amount of data needed for system construction and the lack of sample representation of single center data should not be ignored. To realize the clinical application, these limitations should be addressed in follow-up studies.

### Intelligent Analysis and Disease Diagnosis by Fetal Echocardiography

Fetal echocardiography is challenging because the fetal heart is complex and small, and the fetal heartbeat is very fast. The diagnosis of heart disease in fetuses mainly relies on the experience of the sonographer and overall observations. Presently, AI is useful in the acquisition of fetal heart volume, atrioventricular recognition, ventricular wall thickness measurement, diseases diagnosis as well as in the establishment of the heart intelligent navigation system. With continuous improvements of the algorithm, the automatic and accurate identification of the fetal heart can be realized. The accurate recognition and segmentation of the cardiac cavities by AI can assist in the detection of congenital heart diseases (CHD) such as hypoplastic left heart syndrome, endocardial cushion defects, and large atrial/ventricular septal defects. A recent big data research developed an ensemble of neural networks to identify recommended cardiac views and diagnose complex CHD, which achieved a 95% sensitivity and 96% specificity. This research also demonstrated that the classifier made the decisions based on clinically relevant image features, and overcoming the problems of lack of experience as well as the poor quality of images were the key points in AI-aided diagnosis of CHD (36). However, artifacts, contour loss, noise, and uneven intensities always affect the feature recognization and analysis of fetal heart images (37). Artificial intelligence technique is expected contribute to the standardization and optimization of fetal echocardiography (38).

Image quality and quality control were essential factors for the evaluation of fetal echocardiography. Previous studies have used various algorithms to achieve accurate segmentation and recognition of the structure of the fetal heart, no matter from images or videos. The visibility, position, orientation, and the viewing plane of the fetal heart in ultrasound images were parameters which required attention (39). Good results of segmentation was the basis of accurate assessment. Xu et al. proposed an algorithm for the segmentation of apical four-chamber view in fetal echocardiography. An end-to-end DW-Net (consisted of DCC and W-Net) was used for accurate location and refining of precise boundaries. This approach was helpful for solving the problems of artifacts, speckle noise, and missing boundaries, and it finally reached a Dice similarity coefficient of 0.827 (40). In Dong et al.'s research, they proposed a deep learning framework based on a basic CNN (segmentation of the four-chamber plane), a deeper CNN (determination of the zoom and gain) and ARVBNet (detection of key structures) for quality assessment of fetal echocardiography. This method could recognize the internal structures of the fetal heart, estimate whether the recognition was true, and provide a score (41).

Although many studies aimed to improve the diagnostic efficiency of CHDs, its abnormal anatomical morphological changes limit the probability, especially for those complex CHDs. One solution was detecting indirect signs, such as assessment of ventricular volumes and the thickness of ventricular walls (42, 43). Another way was to implement more comprehensive detection of anatomical structure. The intelligent fetal heart imaging system was based on spatiotemporal image correlation (STIC), which identifies the fetal arterial duct arch and then gradually realizes the imaging of the fetal heart screening sections. VOCAL software was based on STIC, and it is used to measure the ventricular septal volume by 3D imaging, and the correlation between the ventricular septal volume and the GA was then evaluated (44). For the diagnosis of cardiac malformations, several investigators have developed an AI diagnosis system for fetal heart malformations. The system uses big datasets of confirmed normal and abnormal fetal heart images in combination with cloud computing to detect fetal heart malformations. Another typical example was the software named Fetal Intelligent Navigation Echocardiography (FINE), which utilizes intelligent navigation technology on STIC datasets (45). Once the tags were completed, nine standard fetal echocardiography views were automatically generated and simultaneously. These views included a four-chamber view, a five-chamber view, a section of the left ventricular outflow tract, a short axis of the aorta/section of the right ventricular outflow tract, a section of the three-vessel view, an abdomen/stomach bubble section, an arterial ductal arch section, an aortic arch section, and a superior and inferior vena cava section. Several studies have used FINE technology to arrive at a diagnosis of fetal tetralogy of Fallot combined with pulmonary atresia. In addition, Mindray has developed an intelligent fetal heart imaging system for standard sections of fetal 3D volume measurement data. The system could semi-automatically identify six standard sections commonly used in fetal heart examinations according to the position of the four-chamber reference point, which was input by the user to improve the inspection efficiency. Based on the big database of characteristics of fetal heart structures, it adopted deep learning to construct an intelligent fetal heart structure recognition system that could distinguish different anatomical structures, combine the information with the user's input to determine the search range of the fetal heart structures, identify the positions of the main anatomical structures in the volume measurement data, and generate a standard section of the fetal heart according to the positions of the main anatomical structures. Nevertheless, the current fetal heart intelligent imaging system was still susceptible to interference from the fetus, whose movement cannot be controlled. Therefore, suitable algorithms, matrix probe, and real-time 3D ultrasonography are still required.

### Evaluation and Prediction of Cervical Function

Cervical insufficiency is the main factor leading to recurrent abortion and spontaneous preterm birth in the second trimester. It is also one of the causes of neonatal death. Transvaginal ultrasonography is widely used in the evaluation of cervical function during pregnancy. Through the observation and evaluation of cervical length, cervical funnel formation, and other indicators, the development of cervical insufficiency could be predicted and monitored to some extent. However, the specificity and sensitivity of existing techniques and single indicators, such as cervical length, to predict the risk of preterm birth were low, and some systematic assessment software packages could only obtain semi-quantitative assessment indicators, namely, the risk stratification of preterm birth. The lack of practical tools for the evaluation of cervical function always leads to excessive examinations and overtreatment.

Quantitative analysis of tissue texture in images was the main application point of AI in the evaluation of cervical insufficiency. Investigators from Spain applied the quantitative analysis of cervical texture in the evaluation of cervical tissue changes during pregnancy (46). A total of 18 features were extracted from each ultrasound image and area of interest, and a prediction model of GA based on features from the cervical image was established through data segmentation, feature transformation, and model calculation, which indicated that there was a strong correlation between cervical ultrasound images and GA. Based on the low specificity of cervical length for the assessment of cervical function, these investigators adopted the feature combination learning algorithm based on feature transformation and regression, selected the area of interest in the middle of the labium anterius, established the CTx score, and confirmed that the CTx score of pregnant women with a short cervix and term delivery was higher than that of pregnant women with a short cervix and premature delivery (47). This technique provided support for predicting the risk of premature birth in pregnant women with a short cervix.

In addition to texture analysis, the application of omics to predict premature birth has also been useful. A previous study combined AI, proteomics, metabolomics, and ultrasonography, and used a variety of machine learning technologies, which included deep learning, to predict preterm birth, preterm latency, and neonatal treatment time in the NICU during the second trimester (48). Despite the small sample size, the study confirmed that deep learning had an advantage over other types of machine learning in the processing of complex data in the multifactorial prediction of cervical insufficiency.

### Evaluation of Fetal Weight and Gestational Age

Artificial intelligence technique demonstrated its possibility for the accurate generation of fetal weight, which was mainly based on the gestational weeks and the biparietal diameter, AC, and femur length, especially for extreme fetal weights. Yasunari et al. reported that they developed an AI method for estimating the fetal weight. A neural network architecture was trained by deep learning with a dataset that was consisted of ± 2 standard deviation (SD), ± 1.5 SD, and ± 0 SD categories of the approved standard values of ultrasonic measurements of the fetal weights (49).

Preterm birth is a major global health challenge, which is the leading cause of death in children under 5 years of age. Current methods for estimating fetal GA were often inaccurate. For example, at 20–30 weeks of gestation, the width of the 95% prediction interval around the actual GA is approximately 18–36 days. To solve this problem, Russell et al. developed a machine-learning approach to accurately estimate GA using ultrasound-derived and fetal biometric data. The accuracy of the method was determined by reference to exactly known facts pertaining to each fetus specifically, intervals between ultrasound visits, rather than the date of the mother's last menstrual period. The generalization of the algorithm was shown with data from a different and more heterogeneous population. In the context of two large datasets, they estimated the GA to within 3 days between 20 and 30 weeks of gestation with a 95% CI, using measurements made in a 10-week window spanning the second and third trimesters, hence fetal GA can be estimated in the 20–30 weeks GA window with a prediction interval 3–5 times better than with any previous algorithms (50). This would enable improved management of individual pregnancies and help identify at-risk fetuses more accurately than currently possible. At the population level, the higher accuracy was expected to improve fetal growth charts and population health assessments. Machine learning could circumvent long-standing limitations in determining fetal GA and future growth trajectories without recourse to often inaccurately known information, such as the date of the mother's last menstrual period. Using this algorithm in clinical practice could facilitate the management of individual pregnancies and improve population-level health.

## Discussion and Future Perspectives

The combination of AI and ultrasonography is assisting clinicians in the diagnosis of a variety of conditions and diseases, as it can improve efficiency, reduce the rates of misdiagnosis and missed diagnosis, effectively improve the quality of medical services, and ultimately benefit patients. Presently, significant achievements have been made in the application of AI in the fields of obstetrics and gynecology, but the universality and effectiveness of many models still require further studies. Lots of skills have been investigated in current reports to overcome the dilemma of limited accuracy, such as developing ensemble algorithms (51), using ultrasound videos or time-series information as validation set (52, 53), or considering features in complementary imaging modalities (54), etc. In addition, with the constant optimization and modification of algorithms, not only algorithm engineer, but clinicians should have knowledge so that they can eliminate or standardize subjective bias to avoid misdiagnosis in order to achieve objective, fair, and unified generalization standards.

On the other hand, AI related obstetric ultrasound techniques are gradually playing a part in education and social service. For example, fetal ultrasound telemedicine service can link up the specialist fetal medicine center and the remote obstetric unit, which can provide high quality ultrasound diagnosis and specialist consultation as well as reducing the family costs and journey times significantly (55). Furthermore, this technique was proved to be useful in transnational consultation. Since obstetric ultrasound remains unavailable in many developing countries and rural areas, telemedicine/telediagnostic service can increase access to diagnostic obstetric ultrasound in low-resource setting. It had been proved to have excellent agreement with standard of care ultrasound (56). In obstetrics and gynecology education, VR is becoming a new way of simulation-based ultrasound training, which significantly improves learning efficiency and knowledge retention in fetal ultrasound teaching [such as recognizing fetal brain anomalies on ultrasound imaging (57) or studying fetal development (58)]. We believe that with the development of techniques and interdisciplinary integration, there will be a lot more that AI can offer in the field of obstetric ultrasound.

## Author Contributions

ZC is responsible for the conception of the manuscript and the approval of article. ZL is responsible for the interpretation and article drafting. MD is responsible for the review of the literature. ZW is responsible for the critical revision of article. All authors read and approved the final manuscript.

## Conflict of Interest

The authors declare that the research was conducted in the absence of any commercial or financial relationships that could be construed as a potential conflict of interest.

## Publisher's Note

All claims expressed in this article are solely those of the authors and do not necessarily represent those of their affiliated organizations, or those of the publisher, the editors and the reviewers. Any product that may be evaluated in this article, or claim that may be made by its manufacturer, is not guaranteed or endorsed by the publisher.
